# Postbiotic Potential of Heat-Killed *Lacticaseibacillus paracasei* HP7 in Functional Dyspepsia

**DOI:** 10.4014/jmb.2508.08029

**Published:** 2025-10-28

**Authors:** Daehyeop Lee, Ji-Woong Jeong, Joo-Yun Kim, Jae-Jung Shim, Jae-Hwan Lee

**Affiliations:** R&BD Center, hy Co., Ltd., Yongin-si 17086, Gyeonggi-do, Republic of Korea

**Keywords:** *Lacticaseibacillus paracasei*, heat-killed probiotics, functional dyspepsia, postbiotics, exopolysaccharides (EPS), surface-layer proteins (SLPs)

## Abstract

Functional dyspepsia (FD) is a complex gastrointestinal disorder involving impaired motility and digestive dysfunction. This study investigated the postbiotic potential of heat-killed *Lacticaseibacillus paracasei* HP7 (HP7) in a loperamide-induced FD mouse model. Oral administration of heat-killed HP7 improved gastric emptying, and upregulated expression of genes related to smooth muscle contraction. Heat-killed HP7 treatment also modulated key gastrointestinal hormones, including gastrin (GAS), glucose-dependent insulinotropic peptide (GIP), and peptide YY (PYY), and restored the activities of digestive enzymes such as amylase and trypsin. To investigate potential mechanisms, heat-killed HP7 and exopolysaccharides (EPS) and surface-layer proteins (SLPs) derived from HP7 were applied to LPS-treated Caco-2 cells. These functional components strengthened epithelial barrier function by upregulating tight junction-related genes and reducing the expression of inflammatory genes. These results suggest that heat-killed HP7 and its functional cell surface molecules promoted gastric motility, strengthened barrier integrity, and mitigated inflammation through complementary pathways, indicating their potential as safe and effective postbiotic interventions for managing FD.

## Introduction

Functional dyspepsia (FD) is a common gastrointestinal disorder originating from the gastroduodenal region, characterized by symptoms without an identifiable structural cause. The Rome IV classification divides FD into two forms: postprandial distress syndrome (PDS), which involves early satiety and fullness after meals, and epigastric pain syndrome (EPS), which is characterized by burning discomfort and pain in the upper abdomen [1]. FD affects individuals across all age groups and has been shown to reduce quality of life and increase healthcare costs [2]. Despite widespread prevalence, the pathophysiology of FD remains poorly defined and is thought to involve multiple factors, including gastric motility disturbance, visceral hypersensitivity, *Helicobacter pylori* infection, gut-brain axis disturbance, mental and social factors, and mucosal inflammation in the duodenum [3]. Current treatment strategies for FD, including *H. pylori* eradication, proton pump inhibitors (PPIs), prokinetic agents, and 5-HT receptor agonists, often impose additional economic and psychological burdens on patients. Moreover, the majority of patients derive only limited symptomatic benefit from these approaches [4, 5]. These limitations have led to the investigation of alternative therapeutic strategies with improved safety and applicability in long-term management.

Probiotics, defined as live microorganisms that confer health benefits to the host, have been studied as potential modulators of gut microbiota and gastric motility to alleviate FD symptoms [6-8]. Although probiotics offer well-established health benefits, their use is often limited by concerns regarding safety, viability, and storage stability. As a result, non-viable probiotics, such as heat-killed strains commonly referred to as postbiotics, are being explored as safer and more standardized alternatives [9]. Notably, even after thermal inactivation, certain probiotics can retain their functional characteristics, including immunomodulatory and barrier-enhancing effects. Heat treatment disrupts the bacterial cell membrane and facilitates the release of intracellular and surface-associated bioactive components, such as peptidoglycans, lipoteichoic acids, and exopolysaccharides. These molecules, due to their relatively small size and solubility, can more easily penetrate the intestinal mucus layer and interact directly with intestinal epithelial or immune cells, thereby contributing to host health benefits [10].

*Lacticaseibacillus paracasei* HP7 (HP7), a probiotic strain isolated from kimchi, has shown anti-*H. pylori* activity and beneficial effects on gastric function in previous studies [11, 12]. Notably, our prior research demonstrated that live HP7 alleviated FD symptoms in a loperamide-induced mouse model by enhancing gastric emptying and modulating digestive hormones and enzymes [12]. However, the effect of heat-killed HP7 on FD symptoms has not been explored. Furthermore, no studies have examined whether HP7-derived cell surface components, such as EPS and SLPs, contribute to the improvement of FD symptoms. This study evaluated the efficacy of heat-killed HP7 in a loperamide-induced FD mouse model and investigated whether its exopolysaccharides (EPS) and surface-layer proteins (SLPs) restore epithelial barrier function and suppress inflammatory signaling in LPS-challenged Caco-2 cells.

## Materials and Methods

### Preparation of Heat-Killed Probiotics

*L. paracasei* HP7 (HP7) was cultured in Man-Rogosa-Sharpe broth (Difco Laboratories, USA) at 35°C for 24 h. Following incubation, the cells were harvested by centrifugation at 3,000 ×*g* for 15 min, and the supernatant was discarded. Obtained HP7 pellets were washed with sterile phosphate-buffered saline (PBS) twice. The bacterial suspension was subjected to boiling for 20 min to inactivate the cells, yielding heat-killed HP7. Before the bacteria were heat-killed, we measured colony forming units (CFU) using the serial dilution method.

### Animals Experiments

Six-week-old male BALB/c mice were purchased from Dooyeol Biotech (Republic of Korea) and maintained in a controlled environment at a temperature of 20–22°C, a relative humidity of 40–60%, and a 12 h light/dark cycle. All mice were allowed ad libitum access to water and a standard rodent diet (composition as follows: crude protein 18.4%, fat 6.5%, carbohydrates 44.2%, crude fiber 3.8%, neutral detergent fiber 14.7%, and ash 5.5%; Envigo, USA) for 1 week of acclimation.

The mice were randomly divided into five groups with eight mice per group: NOR, non-treatment of loperamide injection; CONT, loperamide injection (10 mg/kg); MOS, loperamide injection + mosapride (3 mg/kg/day, positive control); HP7-L, loperamide injection + a low dose of heat-killed *L. paracasei* HP7 (1 × 10^8^ CFU/kg/day); HP7-H, loperamide injection + a high dose of heat-killed *L. paracasei* HP7 (1×10^9^ CFU/kg/day).

The saline and samples were orally administered once a day for 4 weeks. The mice were fasted for 20 h on the final day of the experiment, and a single intraperitoneal (IP) injection was performed on all mice except the mice in the normal group. The mice were euthanized using carbon dioxide (CO_2_) gas in a chamber. After that, the stomach, small intestine, and blood were collected for further analysis. The stomach tissue was immediately weighed and blood samples were centrifuged at 2,000 ×*g* for 15 min at 4°C to separate serum. All samples were stored at -80°C until analysis. The animal study was conducted according to the guidelines of hy Co., Ltd. (Republic of Korea) and approved by the Institutional Animal Care and Use Committee of hy Co., Ltd. (approval number, AEC-2025-0001-Y). All *in vivo* data for this study derive from a distinct animal cohort conducted under IACUC approval AEC-2025-0001-Y, with unique IDs and batch records; no panels reuse prior datasets.

### Determination of Stomach Weight and Gastric Emptying Using Phenol Red Solution

Phenol red solution (500 μl per mouse), consisting of 0.05% phenol red dissolved in distilled water with 1.5%sodium carboxymethyl cellulose, was orally administered 30 min after intraperitoneal injection of loperamide. The mice were sacrificed after 30 min, and stomach tissue was extracted and weighed immediately.

To evaluate gastric emptying, the absorbance of maintained phenol red in stomach was measured. The collected stomachs were homogenized in 5 ml of 0.1 N sodium hydroxide (NaOH) solution and 0.5 ml of 20% (w/v) trichloroacetic acid (TCA). The mixture was centrifuged at 700 ×*g* for 20 min, and 1 ml of supernatants were added into 4 ml of 0.5 N NaOH. The absorbance was measured at 560 nm using a BioTek Synergy HTX multimode reader (Agilent Technologies, USA).

The gastric emptying value was calculated according to the following formula:

$$\text{Gastric emptying}\left(\%\right)=\frac{1-X}{Y}\times 100$$

X: Absorbance of phenol red solution remaining in the stomach

Y: Absorbance of naïve phenol red mixed with sodium hydroxide.

### Gene Expression Analysis by Quantitative Real-Time Polymerase Chain Reaction (qRT-PCR) in Mouse Tissues

To evaluate the gene expression in response to loperamide-induced functional dyspepsia, qRT-PCR was performed. Total RNA was extracted from the stomach tissue using the Easy-spin Total RNA Extraction Kit (iNtRON Biotechnology, Republic of Korea). Complementary DNA (cDNA) was synthesized from 2 μg of RNA using an Omniscript Reverse Transcription Kit (Qiagen, Germany). And the synthesized cDNA was analyzed using qRT-PCR with TaqMan Gene Expression Assays (Applied Biosystems). The genes used in this study were as follows: glyceraldehyde-3-phosphate dehydrogenase (*GAPDH*, Mm99999915_g1), 5-hydroxytryptamine (serotonin) receptor 4 (*5HT4R*, Mm00434129_m1), anoctamin-1 (*ANO1*, Mm00724407_m1), ryanodine receptor 3 (*RYR3*, Mm01328421_m1), smooth muscle cell myosin light chain kinase (*smMLCK*, Mm00653039_m1), tight junction protein 1 (*TJP1*, Mm01320638_m1), occludin (*OCLN*, Mm00500910_m1), claudin 1 (*CLDN1*, Mm01342184_m1), nuclear factor of kappa light polypeptide gene enhancer in B cells 1 (*NF-κB*), tumor necrosis factor (*TNF-α*, Mm0043258_m1), and interleukin 1 beta (*IL-1β*, Mm00434228_m1). The relative mRNA expression levels were calculated by the 2^-ΔΔCT^ method, data were normalized to *GAPDH*.

### Determination of GI Regulatory Hormones and Digestive Enzyme Activity

The level of regulatory hormones such as gastrin (GAS, CSB-E12924; CUSABIO, USA), gastric inhibitory peptide (GIP, CSB-E08486m), and peptide YY (PYY, CSB-EL019128MO) in the serum were quantified according to the manufacturer’s instructions.

Digestive enzyme activity including amylase, lipase, and trypsin in the small intestine tissue was evaluated. The tissue was homogenized in 1 ml of PBS. The homogenates were centrifuged at 3,000 ×*g* for 20 min and the supernatants were collected. The activity of amylase (ab102523, Abcam, UK), trypsin (ab102531), and lipase (ab102524) was determined according to the manufacturer’s instructions.

### Isolation of Exopolysaccharides (EPS) and Surface-Layer Proteins (SLPs)

For EPS extraction, cultured HP7 in MRS broth for 24 h was centrifuged at 10,000 ×*g* for 10 min at 4°C to collect the supernatants. Trichloroacetic acid (TCA, final concentration 10% w/v) was added, and incubated with shaking (200 rpm) for 24 h at 4°C. After centrifugation, the supernatant was mixed with ethanol (1:2 v/v) and kept at 4°C for 24 h to precipitate EPS. The precipitate was collected by centrifugation, resuspended in DPBS, and dialyzed against PBS using a SpectraPor membrane (MWCO 12-16 kDa; Thermo Fisher Scientific, USA) for 24 h. The obtained EPS was lyophilized and stored at -80°C until use.

SLPs were extracted from HP7 according to the method previously reported by [13].

### Caco-2 Cell Culture Conditions and Samples Treatment

Caco-2 cells were purchased from the Korean Cell Line Bank (Republic of Korea). Cells were cultured in Dulbecco’s modified Eagle’s medium (DMEM) with 1% of penicillin-streptomycin (P/S) and 10% of heat-inactivated fetal bovine serum (FBS) in a humidified atmosphere containing 5% CO_2_ at 37°C. Cells were fully differentiated for 21 days, and the growth medium was changed every 2-3 days. After differentiation, the cells were treated at a concentration of heat-killed HP7 (10^6^ and 10^7^ CFU/ml), EPS (1, and 10 μg/ml) or SLPs (1, and 10 μg/ml) and incubated for 24 h at 37°C. Subsequently, 1 μg/ml of lipopolysaccharide (LPS) was added to the cells for 24 h.

### Gene Expression Analysis by qRT-PCR in Caco-2 Cells

Total RNA was extracted from LPS-stimulated Caco-2 cells and cDNA was synthesized using the same process as mentioned above. The genes used in *in vitro* study were as follows: glyceraldehyde-3-phosphate dehydrogenase (*GAPDH*, Hs02786624_g1), tight junction protein 1 (*TJP1*, Hs01551871_m1), occludin (*OCLN*, Hs05465837_g1), claudin 1 (*CLDN1*, Hs00221623_m1), myosin light chain kinase (*MLCK*, Hs00364926_m1), nuclear factor kappa B subunit 1 (*NF-κB*, Hs00765730_m1), tumor necrosis factor (*TNF-α*, Hs00174128_m1), interleukin 6 (*IL-6*, Hs00174131_m1), and interleukin 1 beta (*IL-1β*, Hs01555410_m1). The relative mRNA expression levels were normalized to *GAPDH* after calculated by the 2^-ΔΔCT^ method.

### Statistical Analysis

The data are expressed as mean ± standard deviation (SD). For multi-group comparisons, one-way ANOVA followed by Tukey’s post hoc test was used. Analyses were performed in GraphPad Prism version 6 (GraphPad, USA). Unless otherwise specified, two-sided *p* < 0.05 was considered statistically significant.

## Results

### Effects of Heat-Killed HP7 on Stomach Weight and Gastric Emptying

**Fig. 1:**
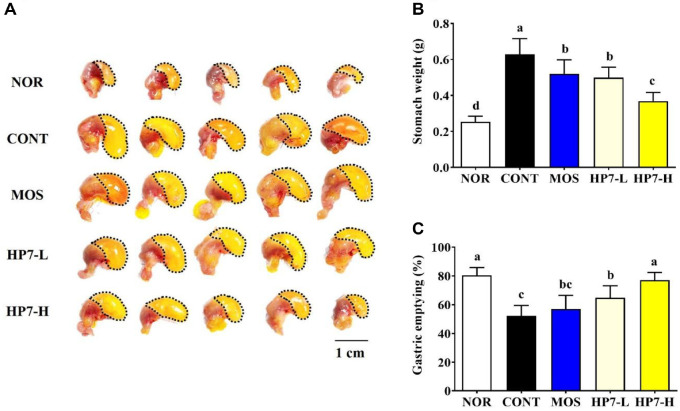
Effects of heat-killed *Lacticaseibacillus paracasei* HP7 on gastric emptying in a loperamide-induced mouse model. (**A**) Representative images of the stomach, (**B**) stomach weight, and (**C**) gastric emptying ratio were evaluated. Data are presented mean ± standard deviation (SD). Different letters indicate significant differences (*p* < 0.05). NOR, normal group; CONT, loperamide injected group; MOS, mosapride-treated group; HP7-L, low-dose heat-killed HP7 treated group (10^8^ CFU/kg/day); HP7-H, high-dose heat-killed HP7 treated group (10^9^ CFU/kg/day).

As shown in the representative images, loperamide injection markedly delayed gastric emptying and increased stomach weight compared to the NOR group (Fig. 1A). The stomach weight in the NOR group was 0.253 g, whereas loperamide injection significantly increased it to 0.629 g. Mosapride treatment significantly reduced the stomach weight to 0.520 g. The stomach weight of HP7-L and HP7-H groups was significantly decreased to 0.498 g and 0.369 g, respectively (Fig. 1B). These findings were supported by the results of the gastric emptying ratio evaluated using remaining phenol red in the stomach (Fig. 1C). As shown in Fig. 1C, gastric emptying ratio in NOR group was 80.37%. In contrast, it was significantly decreased to 52.23% in the CONT group, indicating impaired gastric emptying ability due to loperamide injection. Administration of mosapride and heat-killed HP7 effectively restored gastric emptying function, and the heat-killed HP7 treatment group showed dose-dependent improvement of the gastric emptying ratio. The gastric emptying ratio was 56.92% in the MOS group, and HP7-L and HP7-H groups showed a significant increase in the ratio to 64.87% and 77.07%, respectively.

### Effects of Heat-Killed HP7 on mRNA Expressions of Genes Related to Smooth Muscle Contraction, Tight Junctions, and Inflammation in the Loperamide-induced Mouse Model

**Fig. 2:**
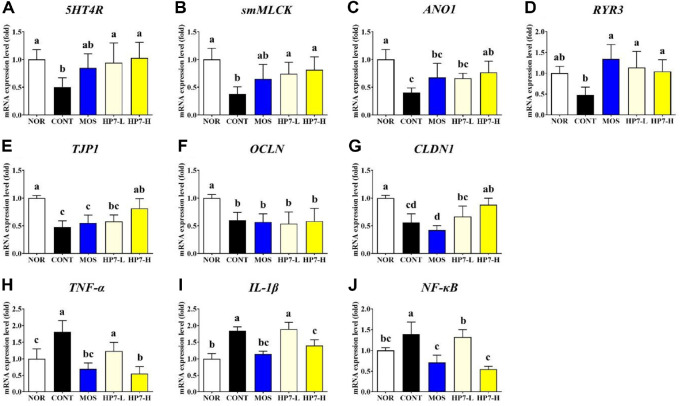
Effects of heat-killed HP7 on gene expression in a loperamide-induced mouse model. The mRNA expression levels of smooth muscle contraction-related genes (**A-D**), tight junction-related genes (**E-G**), inflammatory response-related genes (**H-J**) were evaluated. Data are presented mean ± SD. Different letters indicate significant differences (*p* < 0.05). NOR, normal group; CONT, loperamide injected group; MOS, mosapride-treated group; HP7-L, lowdose heat-killed HP7 treated group (10^8^ CFU/kg/day); HP7-H, high-dose heat-killed HP7 treated group (10^9^ CFU/kg/day).

The mRNA expression of smooth muscle contraction-related genes was significantly downregulated by loperamide injection in this study. By loperamide injection, the expression level of *5HT4R* was reduced to 0.51-fold compared to the NOR group. Mosapride treatment restored *5HT4R* expression to 0.85-fold level. When heat-killed HP7 was administered at 10^8^ and 10^9^ CFU/ml concentrations, the gene expression was significantly upregulated by 0.94- and 1.03-fold, respectively, which is similar to the level of the NOR group (Fig. 2A). The expression of *smMLCK* was markedly decreased by loperamide injection to 0.37-fold relative to the NOR group. In the MOS group, the expression of the *smMLCK* gene was restored to 0.65-fold, while it was upregulated to 0.74-fold and 0.81-fold in the HP7-L and HP7-H groups, respectively (Fig. 2B). In addition, loperamide injection significantly suppressed the expression of *ANO1*, reducing their levels to 0.40-fold and 0.47-fold, respectively, compared to the NOR group. Upon treatment with mosapride, the expression of the *ANO1* gene increased to 0.67-fold. Also, administration of heat-killed HP7 significantly elevated *ANO1* expression to 0.66-fold in the HP7-L group and 0.77-fold in the HP7-H group (Fig. 2C). Similarly, *RYR3* expression, which was suppressed to 0.47-fold in the CONT group, was significantly restored to 1.34-fold in the MOS group, and to 1.14- and 1.04-fold in the HP7-L and HP7-H groups, respectively (Fig. 2D).

We also investigated the effects of heat-killed HP7 on the expression of tight junction-related genes. The CONT group exhibited significantly lower expression levels of *TJP1* (0.48-fold), *OCLN* (0.60-fold), and *CLDN1* (0.56-fold) than the NOR group (Fig. 2E-2G). The HP7-H group showed a significant recovery in the expression of *TJP1* (0.82-fold) and *CLDN1* (0.88-fold), whereas mosapride and the lower HP7 dose exhibited a tendency toward recovery without reaching statistical significance.

Finally, the expression of inflammation-related genes, *TNF-α*, *NF-κB*, and *IL-1β*, was significantly upregulated by loperamide injection, reaching 1.84-, 1.39-, and 1.81-fold, respectively, compared to the NOR group (Fig. 2H-2J). Interestingly, mosapride treatment significantly decreased the expression of these genes to 1.14-, 0.71-, and 0.69-fold, respectively. The expression of *IL-1β*, *NF-κB*, and *TNF-α* was significantly downregulated to 1.40-, 0.55-, and 0.55-fold, respectively, in the HP7-H group. In contrast, only *NF-κB* expression was significantly reduced in the HP7-L group, showing no significant impact on the other inflammatory cytokines.

### Effects of Heat-Killed HP7 on GI Regulatory Hormones and Digestive Enzyme Activities

**Fig. 3:**
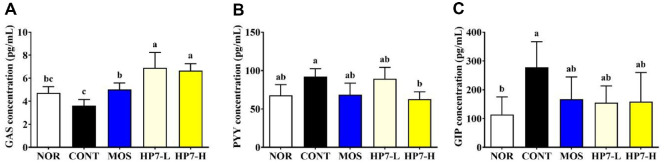
Effects of heat-killed HP7 on digestive hormone levels in the serum of a loperamide- induced mouse model. Serum concentrations of (**A**) gastrin (GAS), (**B**) peptide YY (PYY), (**C**) gastric inhibitory peptide (GIP) were measured. Data are presented mean ± SD. Different letters indicate significant differences (*p* < 0.05). NOR, normal group; CONT, loperamide injected group; MOS, mosapride-treated group; HP7-L, low-dose heat-killed HP7 treated group (10^8^ CFU kg/day); HP7-H, high-dose heat-killed HP7 treated group (10^9^ CFU/kg/day).

Loperamide injection significantly altered the GI regulatory hormones, such as GAS, PYY, and GIP, in serum levels (Fig. 3). The GAS concentration associated with GI motility was significantly reduced from 4.73 pg/ml to 3.61 pg/ml following loperamide injection, but restored to 5.02 pg/ml in the MOS group. The concentration of GAS was markedly increased to 6.90 pg/ml in the HP7-L group and 6.66 pg/ml in the HP7-H group (Fig. 3A). The concentration of PYY, hormone related to suppression of gastric motility, was significantly increased from 67.83 pg/ml of the NOR group to 91.96 pg/ml of the CONT group. The MOS group significantly downregulated the concentration of PYY to 68.49 pg/ml, comparable to the NOR group. The administration of heat-killed HP7 at concentrations of 10^8^ and 10^9^ CFU/ml showed different results. Administration of 10^8^ CFU/ml of heat-killed HP7 did not significantly reduce the concentration of PYY in serum, and it was 89.28 pg/ml. Otherwise, the concentration of PYY in serum was significantly decreased to 62.75 pg/ml in the HP7-H group (Fig. 3B). Likewise, the concentration of GIP, a hormone which inhibits gastric acid secretion and gastric motility, was significantly increased to 278.13 pg/ml by loperamide injection from 114.35 pg/ml of the NOR group. The GIP concentration in serum tended to decrease in all groups, but there was no significant difference compared to the CONT group (Fig. 3C).

**Fig. 4:**
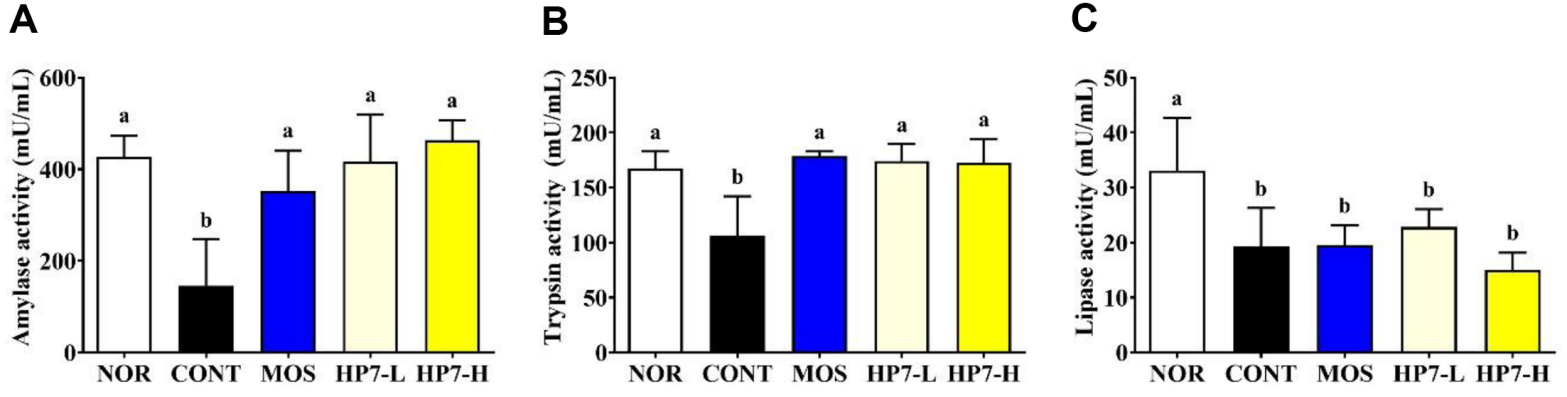
Effects of heat-killed HP7 on activities of digestive enzymes in a loperamide-induced mouse model. The activity of (**A**) α-amylase, (**B**) trypsin, (**C**) lipase were assessed. Data are presented mean ± SD. Different letters indicate significant differences (*p* < 0.05). NOR, normal group; CONT, loperamide injected group; MOS, mosapride-treated group; HP7-L, low-dose heat-killed HP7 treated group (10^8^ CFU/kg/day); HP7-H, high-dose heat-killed HP7 treated group (10^9^ CFU/ kg/day).

The activities of digestive enzymes, including α-amylase, trypsin, and lipase, were significantly reduced by loperamide injection (Fig. 4). The α-amylase activity in the CONT group was decreased to 146.15 mU/ml from 427.52 mU/ml in the NOR group. In contrast, the α-amylase activity was increased to 352.67 mU/ml by the mosapride treatment. Notably, the α-amylase activity was elevated to 416.26 mU/ml in the HP7-L group and further increased to 463.84 mU/ml in the HP7-H group (Fig. 4A). In a similar trend, the trypsin activity reached 178.56 mU/ml with mosapride treatment, and 174.22 and 172.64 mU/ml with low and high doses of administering heat-killed HP7, respectively, showing significant upregulation compared to the CONT group (Fig. 4B). Meanwhile, lipase activity exhibited a distinct pattern compared to other enzyme activities. It declined from 33.12 mU/ml in the NOR group to 19.32 mU/ml after loperamide injection. Unlike the other digestive enzymes, lipase activity was not significantly restored by either mosapride or heat-killed HP7 treatment (Fig. 4C).

### Effect of Heat-killed Bacteria and Its-Derived EPS and SLPs on mRNA Expression of Genes Related to Tight Junctions and Inflammation in the LPS-Induced Caco-2 Cell Model

**Fig. 5:**
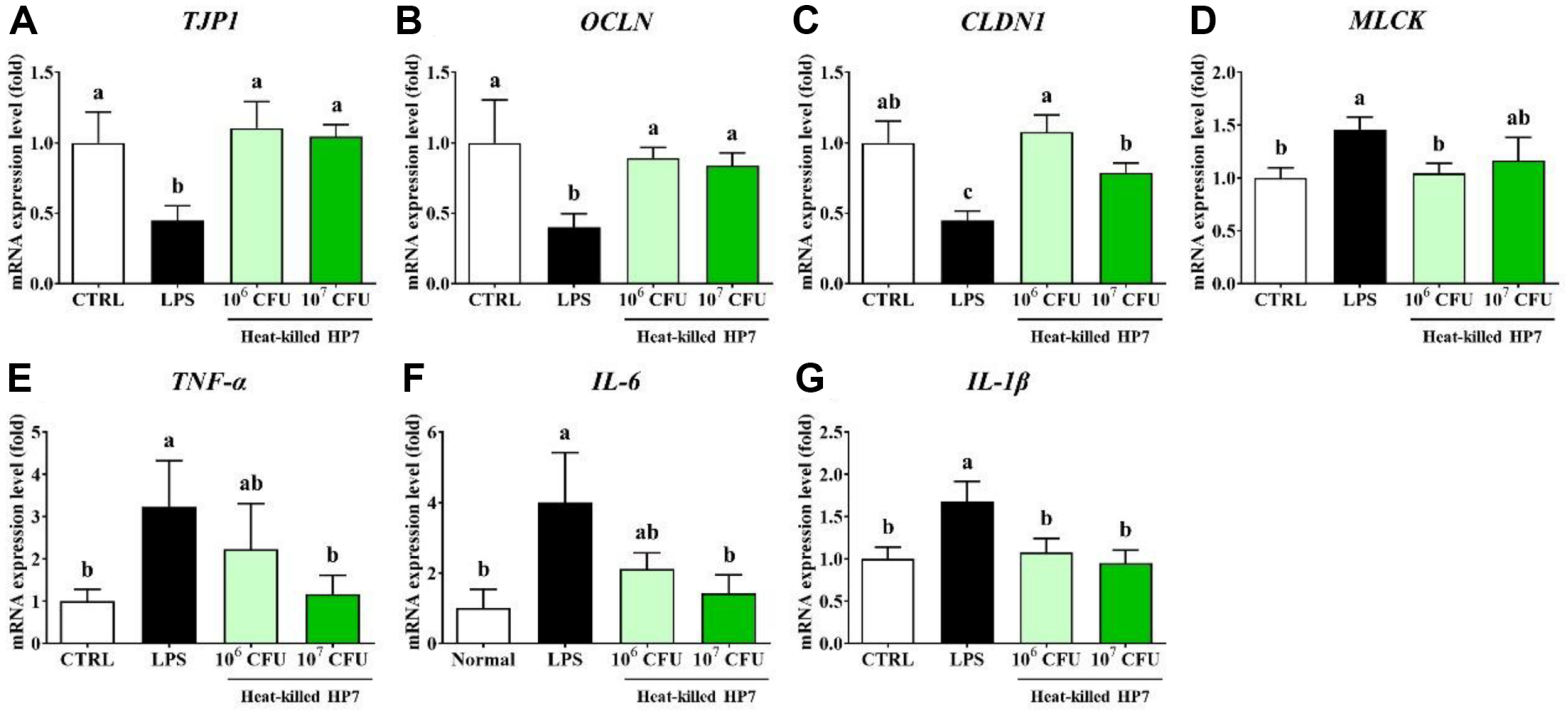
Effect of heat-killed HP7 on gene expression in lipopolysaccharide (LPS)-induced Caco-2 cell model. The mRNA expression levels of tight junction-related genes (**A-D**), and inflammatory cytokines (**E-G**) were evaluated. Data are presented mean ± SD. Different letters indicate significant differences (*p* < 0.05).

To further support the results from the animal experiments, we investigated the gene expression related to tight junction integrity and inflammation, factors implicated in FD, using a Caco-2 cell model. FD-like conditions were induced by treating Caco-2 cells with LPS to induce epithelial barrier disruption and inflammatory responses, and the restoration effect of heat-killed HP7 was evaluated. As shown in Fig. 5, treatment with heat-killed bacteria significantly modulated the expression of genes associated with tight junction integrity and inflammation in the LPS-induced Caco-2 cell model. LPS stimulation led to a marked decrease in the expression of tight junction integrity genes, including *TJP1* (0.45-fold), *OCLN* (0.40-fold), and *CLDN1* (0.45-fold), and a significant increase in the expression of *MLCK* (1.46-fold), tight junction permeability genes (Fig. 4A-4D). However, treatment with heat-killed HP7 notably restored the expression of these genes. Interestingly, low-dose (10^6^ CFU/ml) treatment was more effective than the high-dose (10^7^ CFU/ml) in restoring the expression of tight junction genes in this study. Additionally, LPS treatment significantly increased the expression of inflammatory cytokines, including *TNF-α* (324%), *IL-6* (401%), and *IL-1β* (168%) compared to the control. Treatment with heat-killed HP7 markedly downregulated the expression of these cytokines in a dose-dependent manner (Fig. 4E-4G).

After isolation of HP7-derived EPS or SLPS, the cell viability was evaluated with MTT using Caco-2 cells (Data not shown). The cell viability was 94.7%-104.4% in EPS treatments and 98.7%-110.3% in SLPs treatments, respectively. Based on these results, the expression of genes related to FD improvement was evaluated in the LPS-induced Caco-2 cell model.

**Fig. 6:**
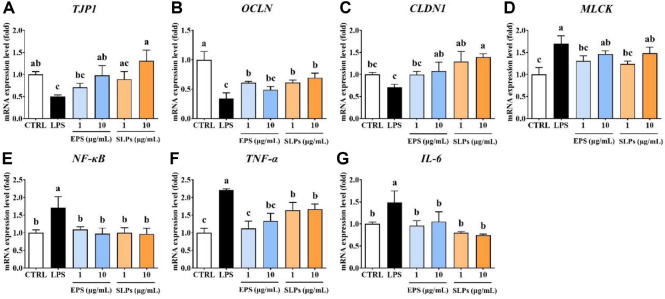
Effect of exopolysaccharides (EPS) and surface-layer proteins (SLPs) isolated from *L. paracasei* HP7 on gene expression in LPS-induced Caco-2 cell model. The mRNA expression levels of tight junction-related genes (**A-D**), and inflammation response-related genes (**E-G**) were evaluated. Data are presented mean ± SD. Different letters indicate significant differences (*p* < 0.05).

The yields of EPS and SLPs were 1 g/l and subsequently analyses were conducted using appropriately diluted concentrations based on their respective yields. After that, we evaluated the expression of genes related to tight junctions and inflammation using EPS and SLPs, which are functional components of heat-killed HP7. The expression levels of tight junction integrity-related genes, *TJP1* (0.50-fold), *OCLN* (0.34-fold), and *CLDN1* (0.71-fold), were significantly lower following LPS treatment compared to the control (Fig. 6A-6C). HP7-derived EPS or SLPs treatment exhibited a increase in these three gene levels. The gene *MLCK* which regulates intestinal epithelial permeability, was significantly upregulated by LPS treatment to 1.70-fold, indicating that the tight junction permeability was increased in Caco-2 cells. Treatment with EPS or SLPs showed downregulation of *MLCK* expression (Fig. 6D). These results suggest that HP7-derived components could be helpful to restore tight junction integrity and reduce permeability. Also, the expression of inflammation-related genes, including *NF-κB*, *TNF-α*, and *IL-6*, were elevated to 1.71-, 2.21-, and 1.49-fold by LPS stimulation in the Caco-2 cell model (Fig. 6E-6G). In contrast, treatment with HP7-derived EPS or SLPs led to the downregulation of inflammation-related gene expression, with *NF-κB* expression significantly restored to the normal level. The expression of *TNF-α* was significantly suppressed by both EPS and SLPs treatments. The expression of *TNF-α* was reduced to 1.12-fold when 1 μg/ml EPS was treated and to 1.64-fold at the same concentration of SLPs treatment. Upregulated *IL-6* expression by LPS was significantly reduced by 1 μg/ml EPS treatment, bringing its expression down to 0.96-fold. SLPs treatment also significantly suppressed *IL-6* expression in a dose-dependent manner, with 1 μg/mL and 10 μg/mL reducing it to 0.80- and 0.75-fold, respectively.

## Discussion

The etiology and pathophysiology of FD remain poorly understood, but are widely believed to be multifactorial. Although the efforts to alleviate FD symptoms have received increasing attention [14, 15], few studies have investigated the potential of heat-killed bacteria in improving FD symptoms. In our previous study, the live form of *L. paracasei* HP7 exhibited potential in relieving FD by modulating intestinal peristalsis and digestive factors [12]. However, due to the intrinsic limitations associated with the viability and stability of live probiotics [16], heat-killed probiotics offer several advantages. They are safer, eliminating the risk of infection, and their enhanced stability facilitates formulation into functional foods, nutraceuticals, or capsule-based products [17]. Furthermore, the global heat-killed probiotics market was valued at USD 1.32 billion in 2024 and is projected to reach USD 3.54 billion by 2033 [18]. Accordingly, this study aimed to evaluate the therapeutic potential of heat-killed HP7 in a loperamide-induced FD mouse model.

Loperamide, a μ2-opioid receptor agonist, was used to induce FD symptoms by inhibiting the activity of the GI myenteric plexus, thereby reducing the contractile tone of the circular and longitudinal smooth muscles [19]. This suppression of GI motility closely resembles the delayed gastric emptying commonly observed in FD patients, making the loperamide model suitable for evaluating prokinetic agents or gut-modulatory interventions. Nevertheless, FD involves not only impaired gastric motility but also visceral hypersensitivity, altered gut-brain interactions, and psychosocial factors. Thus, while the loperamide model is useful for studying motility-related mechanisms, it does not fully explain the complexity of FD, and this limitation should be considered when interpreting the findings. Mosapride, a selective 5-hydroxytryptamine 4 (5-HT4) receptor agon used as a positive control, enhances GI motility and promotes gastric emptying by stimulating acetylcholine release [20]. All *in vivo* data were obtained from a distinct animal cohort under IACUC approval, and no data were reused from previous experiments.

Our results indicated that loperamide injection notably delayed gastric emptying, as evidenced by the visual enlargement of the stomach, the increased stomach weight, and the residual phenol red solution, consistent with previous findings [12, 21]. Given that the delayed gastric emptying is a representative feature of FD, these results may have been attributed to the loperamide injection. This delayed gastric emptying was effectively ameliorated by administration of mosapride and heat-killed HP7 at 10^8^ or 10^9^ CFU/ml. The mosapride group exhibited a significantly lower stomach weight, and the heat-killed HP7 groups showed a dose-dependent reduction in stomach weight. Similarly, mosapride treatment increased the gastric emptying ratio, and heat-killed HP7 treatments dose-dependently improved gastric emptying. In summary, these results suggest that heat-killed HP7 could contribute to ameliorating FD symptoms by improving delayed gastric emptying.

The four genes associated with smooth muscle contraction, such as *5HT4R*, *smMLCK*, *ANO1*, and *RYR3*, supported the results of improved gastric emptying. In this study, both mosapride and heat-killed HP7 significantly restored the downregulated *5HT4R* gene expression by loperamide injection. 5HT and its receptors contribute to the regulation of smooth muscle contraction, and mosapride, a 5HT4R agonist, is mainly used to treat functional disorders with impaired GI motility [22]. This result suggests that heat-killed HP7 may exert a prokinetic effect on the GI tract, similar to mosapride. In addition, heat-killed HP7 treatment significantly upregulated the expression of GI motility-related genes, including *smMLCK*, *ANO1*, and *RYR3*, which are suppressed by loperamide injection. Among these, *ANO1* and *RYR3* are key regulators of intracellular Ca^2+^ signaling in interstitial cells of Cajal (ICCs), essential for generating electrical slow waves in the GI tract [23]. These Ca^2+^ signals propagate to adjacent smooth muscle cells via gap junctions, inducing membrane depolarization and promoting contraction through *smMLCK* activation [24]. Therefore, the restoration of these gene expression suggests that heat-killed HP7 may improve GI motility by enhancing smooth muscle contractility. However, there is a lack of c-Kit analysis to directly evaluate the function of ICCs. As ICCs dysfunction is closely associated with delayed gastric emptying and dyspeptic symptoms, future studies incorporating ICCs marker will be necessary to clarify whether heat-killed HP7 exerts its effect through modulation of ICCs activity.

Recent studies reported the duodenal permeability and immune cell infiltration in FD patients [25]. Also, the level of *TNF-α*, *IL-6*, and *IL-1β* in the serum of FD patients could activate mast cells in the gastric mucosa, thereby contributing to impaired GI motility and altered visceral sensation [26]. Therefore, we evaluated the effect of heat-killed HP7 on tight junction- and inflammation-related genes. Previous studies have indicated that loperamide induces intestinal barrier dysfunction by suppressing tight junction-related genes, and triggers inflammatory response by upregulating inflammation-related genes [27, 28]. Consistently, in our study, loperamide injection induced tight junction disruption via downregulating the expression of tight junction-related genes, including *TJP1*, *OCLN*, and *CLDN1*, and triggered an inflammatory response through the upregulation of inflammation-related genes, such as *TNF-α*, *IL-1β*, and *NF-κB*. Administration of a high dose of heat-killed HP7 positively regulated the expression of these genes, suggesting its potential to ameliorate FD by modulating epithelial barrier function and inflammation. However, the lack of detailed signaling pathway analyses, such as NF-κB/MAPK, necessitates further studies to overcome limitations in the mechanistic understanding of how heat-killed HP7 components regulate inflammation and barrier integrity at the molecular level.

We also investigated the effect of heat-killed HP7 on modulating GI regulatory hormones, including GAS, PYY, and GIP, and digestive enzyme activities, including α-amylase, trypsin, and lipase. GAS stimulates gastric acid, bile, pancreatic enzymes, and small intestinal juice secretion [29], and PYY inhibits GI secretion and motility [30]. GIP, also known as glucose-dependent insulinotropic peptide, has been shown to suppress gastrin stimulated gastric acid secretion and delay GI transit in animal models, suggesting its potential contribution to FD symptoms via both endocrine and motility pathway [31-34]. Many studies indicated that hormones are related to gastric dysmotility. As expected, loperamide injection led to a decrease in the concentration of GAS, and an increase in the concentration of PYY and GIP in this study. Administration of heat-killed HP7 significantly modulated the levels of regulatory hormones, exhibiting a pattern similar to that reported in a previous study using the live form of the strain [12].

Digestive enzymes, including amylase, protease, and lipase, are produced and secreted by the GI system and break down large molecules present in food, such as carbohydrates, proteins, and fats, into smaller molecules for easy absorption [35]. Transient deficiency in these enzymes is regarded as one of the contributing factors to FD [36]. Consistent with previous findings, loperamide injection significantly reduced the activities of amylase, trypsin, and lipase [37]. Unlike amylase and trypsin, lipase activity was not restored in our study. Lipase requires the presence of bile acids for emulsification of dietary fat and is regulated by cholecystokinin (CCK)-mediated pathways [38]. In FD models, delayed gastric emptying and impaired CCK responses may reduce bile flow and pancreatic enzyme secretion, thereby limiting lipase activity recovery. Although further studies are needed, it is plausible that heat-killed HP7 are unlikely to directly modulate bile secretion or CCK-mediated signaling. Meanwhile, mosapride is commonly used as a prokinetic agent for managing FD, there is no direct evidence that it modulates the expression or activity of digestive enzymes. Subsequently, the recovery of enzyme activities following mosapride administration is likely attributed to the improvement of GI motility rather than a direct stimulatory effect on enzyme production or secretion.

To investigate the mechanisms responsible for the *in vivo* efficacy of heat-killed HP7, we conducted a series of *in vitro* experiments using a Caco-2 cell model. Patients with FD, particularly those experiencing epigastric symptoms, have been reported to exhibit increased intestinal permeability, especially in the proximal small bowel or duodenum, which is accompanied by mucosal immune activation and symptoms [39]. Impaired epithelial integrity is considered a potential pathogenic mechanism contributing to low-grade duodenal inflammation and consequently symptoms of FD [40, 41]. Therefore, we examined the effects of heat-killed HP7, bacteria itself, on tight junction integrity and inflammatory responses. Based on these results, we further isolated functional cell surface components, including exopolysaccharides (EPS) and surface-layer proteins (SLPs) from HP7, and evaluated their individual contributions to epithelial barrier function and inflammation in the LPS-stimulated Caco-2 cell model.

The expression of the tight junction integrity-related genes, *TJP*, *OCLN*, and *CLDN1*, was significantly decreased, while the tight junction permeability-related gene, *MLCK*, was significantly increased by LPS treatment. Additionally, the expression of inflammatory cytokines, including *TNF-α*, *IL-6*, and *IL-1β*, were significantly upregulated compared to non-treated condition. Treatment with heat-killed bacteria restored gene expression to normal levels and contributed to the restoration of epithelial barrier function. The effects of bacterial surface components on the regulation of these genes were also evaluated. EPS derived from lactic acid bacteria (LAB) have been reported to exert beneficial effects on the GI tract intact, by interacting with intraluminal water to protect mucosal barrier disruption [42, 43]. Similarly, SLPs, which are surface-associated components, promote bacterial adhesion to intestinal epithelial cells and are implicated in host-microbe interactions, particularly immune modulation [44]. As expected, treatment with its functional components, such as EPS and SLPs, markedly enhanced tight junction integrity by upregulating tight junction-related genes, and suppressed the expression of inflammation-related genes. These results suggest that the HP7 postbiotics could attenuate inflammatory responses, while also reinforcing epithelial barrier integrity, indicating their therapeutic potential in managing FD symptoms. However, several limitations should be acknowledged despite these promising findings. First, detailed profiling and *in vivo* validation of HP7-derived EPS and SLPs were not undertaken, which restricts the translation of these results to physiological contexts. Second, although the Caco-2 cell model is a valuable tool, it cannot fully recapitulate the complex pathophysiology of FD and therefore reflects only a portion of the condition. Thus, further profiling and *in vivo* studies are warranted to verify the therapeutic potential of EPS and SLPs and to elucidate the specific signaling pathways in volved in FD.

In summary, this study demonstrated that heat-killed *L. paracasei* HP7 significantly enhanced gastric emptying, as evidenced by the reduction in stomach weight and residual phenol red content. These effects may be mediated through the regulation of genes associated with smooth muscle contraction, tight junction integrity, and inflammatory responses. Additionally, our findings indicated the potential of heat-killed HP7 to alleviate FD symptoms by modulating digestive hormones (GAS, PYY, and GIP), and activities of digestive enzymes (α-amylase and trypsin). Furthermore, heat-killed HP7 and its cell surface components, EPS and SLPs, enhanced tight junction integrity and suppressed the inflammatory response in the LPS-induced Caco-2 model, providing insights into the mechanisms underlying the regulation of FD symptoms. Taken together, these findings suggest that heat-killed HP7 and its cell components represent promising therapeutic candidates for managing FD symptoms, and motivate targeted mechanistic studies and clinical trials to confirm efficacy and safety.
